# Functional Nucleic Acid Nanostructures for Mitochondrial Targeting: The Basis of Customized Treatment Strategies

**DOI:** 10.3390/molecules30051025

**Published:** 2025-02-24

**Authors:** Wanchong He, Siyu Dong, Qinghua Zeng

**Affiliations:** School of Pharmaceutical Sciences and Food Engineering, Liaocheng University, Liaocheng 252059, China

**Keywords:** FNA nanostructures, mitochondria, customized treatment strategy

## Abstract

Mitochondria, as vital organelles, play a central role in subcellular research and biomedical innovation. Although functional nucleic acid (FNA) nanostructures have witnessed remarkable progress across numerous biological applications, strategies specifically tailored to target mitochondria for molecular imaging and therapeutic interventions remain scarce. This review delves into the latest advancements in leveraging FNA nanostructures for mitochondria-specific imaging and cancer therapy. Initially, we explore the creation of FNA-based biosensors localized to mitochondria, enabling the real-time detection and visualization of critical molecules essential for mitochondrial function. Subsequently, we examine developments in FNA nanostructures aimed at mitochondrial-targeted cancer treatments, including modular FNA nanodevices for the precise delivery of therapeutic agents and programmable FNA nanostructures for disrupting mitochondrial processes. Emphasis is placed on elucidating the chemical principles underlying the design of mitochondrial-specific FNA nanotechnology for diverse biomedical uses. Lastly, we address the unresolved challenges and outline prospective directions, with the goal of advancing the field and encouraging the creation of sophisticated FNA tools for both academic inquiry and clinical applications centered on mitochondria.

## 1. Introduction

Mitochondria, often referred to as the “powerhouses” of eukaryotic cells, are responsible for generating adenosine 5′-triphosphate (ATP) via oxidative phosphorylation [1]. Beyond their fundamental role in energy production, mitochondria are integral to cellular homeostasis, contributing to metabolic regulation and responding to both internal and external stressors such as nutrient deficiencies and redox imbalances [1,2]. These organelles also manage the synthesis and removal of numerous metabolic intermediates and waste products [2]. Moreover, mitochondria act as critical regulators of programmed cell death, including apoptosis and necroptosis [3]. For instance, they store pro-apoptotic proteins and control their release into the cytoplasm to trigger apoptotic pathways [4,5]. Dysregulation of mitochondrial functions has been strongly associated with the onset and progression of various diseases, including diabetes, cancer, and neurodegenerative disorders [6]. Consequently, significant efforts are focused on the in situ analysis of specific mitochondrial components to uncover the molecular mechanisms underpinning these processes. Additionally, researchers are translating insights into mitochondrial biology into therapeutic strategies, as growing evidence highlights the superior efficacy of mitochondria-targeted treatments compared to conventional approaches [7,8].

Functional nucleic acids (FNAs), including DNA and RNA aptamers, Triplex DNA, DNAzymes, RNAzymes, DNA tiles, and DNA origami, have seen widespread use as molecular recognition elements for specific binding with various targets [9,10,11,12]. Unlike other substances, FNA offers several distinctive attributes that make it highly compatible with biological systems. Firstly, as an inherent component of life, DNA exhibits excellent biocompatibility. Secondly, FNA can be readily functionalized with diverse chemical groups, such as fluorophores, phosphorothioates, or therapeutic agents, through chemical synthesis or enzymatic modification [13]. Furthermore, the specific biorecognition capabilities of aptamers and DNAzymes have been discovered and characterized [14,15,16]. Notably, the Watson–Crick base pairing mechanism provides FNA with predictable thermodynamic behavior and exceptional programmability, facilitating the design of intricately shaped nanostructures and adaptive nanomachines capable of dynamic reconfiguration in response to biological stimuli [17,18,19]. Despite progress in FNA-based nanobiotechnology for diagnostics and imaging, mitochondria-targeted FNA systems remain underdeveloped [20,21,22,23,24,25,26,27,28,29,30,31,32,33,34]. This limitation primarily stems from the inability of most FNA-based nanostructures to achieve precise spatial and temporal localization within organelles, which is critical for executing specialized functions.

In recent years, researchers have been actively exploring the fusion of DNA-based technologies with organelle-specific nanotechnology to create sophisticated DNA nanodevices capable of precisely targeting mitochondria and performing their designated functions at the subcellular level [35]. Compared with other mitochondria targeting strategies (e.g., peptide-based targeting or lipid nanoparticles (LNPs), FNA nanostructures are highly programmable, biocompatible, and specific, making them ideal for applications requiring precision targeting, such as biosensing and gene editing. Meanwhile, the delivery efficiency also needs to improve by more targeted modification or by combination with the LNP technique [36,37]. This review provides an overview of the latest breakthroughs in DNA-driven nanobiotechnologies aimed at mitochondrial imaging and therapy. The goal of this review is to comprehensively summarize the current mitochondria-targeted strategies based on functional nucleic acid nanostructures, clarify their advantages over other current mitochondrial targeting methods, and explore the current shortcomings and challenges of this method as well as the direction of future improvement and development. Initially, we delve into mitochondria-specific DNA nanosensors designed for selective imaging of essential mitochondrial molecules, such as nucleic acids, enzymes, small molecules, and metal ions. Particular attention is given to the underlying design strategies of these nanosensors, which offer promising tools for investigating the intricate molecular dynamics associated with mitochondrial roles in health and disease. Next, we outline recent advancements in two key DNA-based therapeutic approaches targeting mitochondria. The first involves the use of modularly engineered DNA nanodevices to facilitate the controlled delivery of therapeutic agents into mitochondria, while the second employs in situ programmed DNA self-assembly to directly modulate mitochondrial function. Finally, we examine the existing hurdles and future prospects within this field. By consolidating the latest advancements in this promising area, we aim to inspire the development of innovative methodologies that can benefit both fundamental research and clinical applications involving mitochondria.

## 2. Mitochondria Targeting Strategies Based on FNA Nanostructures

### 2.1. FNA Targeting Mitochondrial RNA (mtRNA)

In eukaryotic cells, while the majority of DNA is tightly packed into chromatin structures and housed within the nucleus, mitochondria possess a unique subset of DNA, known as mtDNA, which distinguishes them from other organelles [38]. Beyond mtDNA, an increasing body of evidence suggests that microRNAs (miRNAs), primarily transcribed from the nuclear genome and typically active in the cytoplasm, can also localize within mitochondria, where they are referred to as mitomiRs [39,40]. These nucleic acids localized in mitochondria play critical roles in regulating mitochondrial functions. For instance, mutations in mtDNA are known to disrupt mitochondrial functionality, potentially causing various metabolic disorders [41]. Furthermore, abnormal expression levels of mitomiRs have been associated with a wide array of diseases, including neurodegenerative conditions, cardiovascular disorders, and cancers [42,43]. Some mitomiRs even hold promise as diagnostic biomarkers or therapeutic targets for these diseases [43]. Despite significant advancements in DNA-based sensing technologies for detecting and imaging nucleic acids in live cells [44,45,46,47], their application in tracking mitochondrial targets presents substantial challenges. These difficulties arise from the lack of mitochondria-specific targeting capabilities. Moreover, many nucleic acid targets, such as miRNAs, are not confined to a specific organelle. Conventional sensing approaches, which rely on an “always active” detection mechanism, risk premature activation upon encountering these targets before reaching the intended organelle, thereby compromising spatial accuracy. FNA nanostructures provide a promising approach for targeting mtDNA mutations through precise sequence recognition, targeted delivery, and controlled gene modulation. Their interaction with mtDNA mutations occurs via several mechanisms: 1. Targeted gene editing and repair. FNA nanostructures can deliver gene editing systems to mitochondria to selectively cleave mutant mtDNA, promoting the replication of wild-type mtDNA. Unlike nuclear DNA editing, mtDNA lacks efficient DNA repair mechanisms, making targeted cleavage a viable approach for shifting heteroplasmy levels. 2. Selective silencing of mutated mtDNA. FNA nanostructures designed as small interfering RNAs or mitochondria-targeted aptamers can selectively bind mutated mt-mRNAs, reducing the expression of defective proteins. Aptamer-functionalized FNAs provide high specificity in targeting mutant transcripts while sparing wild-type sequences. 3. Mitochondrial delivery of therapeutic genes. FNA nanostructures can be engineered as nanocarriers to deliver therapeutic RNA/DNA molecules, compensating for mtDNA mutations.

**Figure 1:**
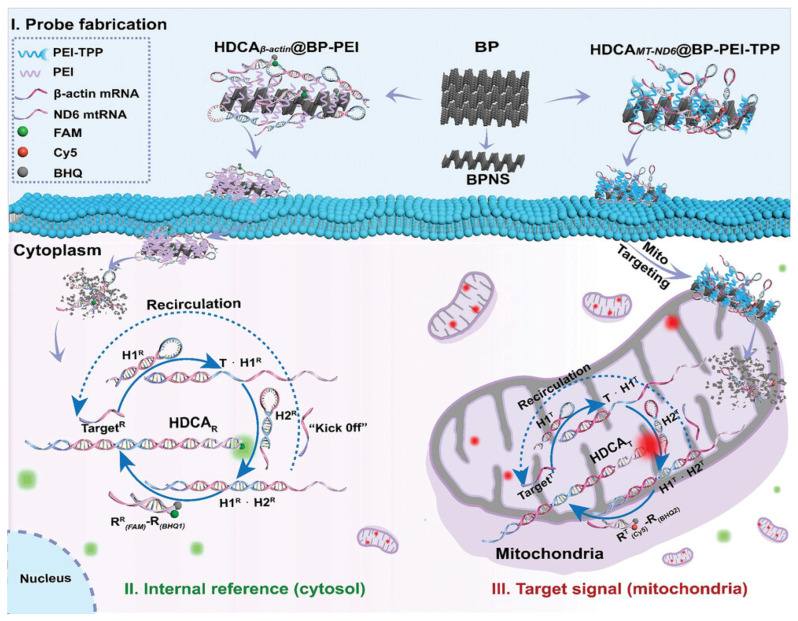
Schematic of the dual-color imaging system for quantitative analysis of specific mtRNA in living cells: (**I**) Fabrication of the HDCA probe onto BPNS-based vehicles for effective delivery to both the cytosol and mitochondria. (**II**) Internal reference module for detecting housekeeping gene (TargetR) in the cytoplasm. (**III**) Reporting module designed to specifically target mitochondrial RNAs (TargetT) [48].

To address the above-mentioned issues, some FNA nanostructures which target mitochondria were developed. The Qian group introduced a novel “RT-qPCR mimic” system, incorporating a signal amplification mechanism without the need for enzymes [48]. This system employs a hairpin DNA cascade amplifier (HDCA), which is composed of two metastable hairpin DNA structures and a hybrid DNA duplex reporter (Figure 1), to independently visualize mtRNA and cytosolic reference mRNA. To enable the precise delivery of probes to subcellular targets such as mitochondria and the cytosol, black phosphorus nanosheets (BPNSs) were utilized. These nanosheets, known for their excellent biocompatibility, high molecular loading efficiency [49,50], and ability to degrade into non-toxic phosphate and phosphonate ions under physiological conditions [50,51,52,53,54], served as stable transport platforms. Additionally, the BPNS surface’s unique combination of a high surface-area-to-volume ratio and periodic atomic grooves offered optimal attachment sites for nucleic acids [55,56,57], shielding them from degradation by endogenous nucleases. Demonstrating the approach, the HDCA designed to detect mtRNA encoding NADH dehydrogenase subunit 6 (mtRNAND6) was loaded onto mitochondria-specific BPNS carriers, BP-PEI-TPP, for targeted delivery and detection. Simultaneously, the levels of β-actin mRNA (referred to as mRNAβ-actin) in the cytoplasm were measured as an internal control. This innovation represents the first dual-color imaging platform capable of accurately quantifying specific mitochondrial RNAs in living cells.

The method employing precise spatial and temporal control serves as a versatile tool for investigating the biological roles of mitomiRs. Building on this targeting strategy, Chen et al. incorporated a fluorescence-encoded error correction system to create a near-infrared (NIR)-responsive DNA platform capable of simultaneously visualizing three mitomiRs associated with the mitochondrial mt-ND1 genome in drug-resistant cells [58]. Furthermore, a nanoscale DNA computing device was engineered to track two mitomiRs during the process of cell apoptosis [59]. More recently, the development of an AIE-labeled DNA probe, integrated with a polymer-based nanocarrier for targeted delivery, enabled the pH-sensitive exonuclease-driven imaging of mitomiRs [60].

**Figure 2:**
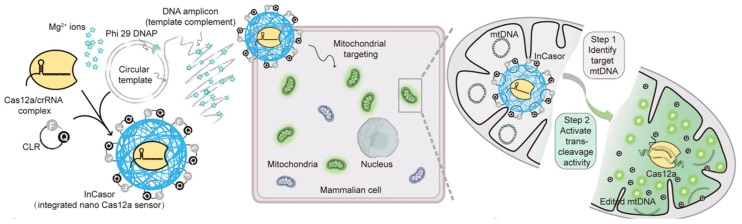
Schematic of the InCasor: This system comprises a DNF, Cas12a/crRNA complex, and a CLR for imaging mtDNA in live cells [61]. Through the strategic design of crRNA, single nucleotide variants in mitochondrial DNA can be precisely recognized. Additionally, increasing the intracellular concentration of Mg^2+^ significantly boosts the collateral trans-cleavage activity of Cas12a, thereby amplifying the detection signal within living cells.

In contrast to miRNAs, mtDNA is confined to the mitochondria and exhibits a relatively stable concentration. However, due to the elevated levels of reactive oxygen species (ROS) within mitochondria and the absence of histone protection, mtDNA is particularly vulnerable to oxidative damage, which can result in pathogenic mutations. Importantly, mtDNA mutations are highly dynamic and dispersed across the entire genome, making the detection of mutations present in low abundance at specific loci particularly challenging. To address this, Zhang et al. recently developed an integrated nanoscale Cas12a sensor (referred to as InCasor). This system employs a DNA/Mg^2+^ hybrid nanoflower (DNF) functionalized with aptamers to deliver Cas12a-related components directly to mitochondria. These components include the Cas12a/crRNA complex and a circular reporter (CLR) labeled with a fluorophore and quencher, enabling the identification of mtDNA mutations in live cells and in vivo (Figure 2) [61].

A significant achievement of this research is the successful mitochondria-specific delivery of the Cas12a/crRNA complex using InCasor. The mitochondrial bilayer membrane poses a significant barrier, making the transport of guide RNA and Cas proteins into mitochondria notoriously difficult [62]. To tackle this obstacle, this study employed a nanoparticle-based delivery approach, a strategy that has proven effective in transporting proteins and nucleic acids into mitochondria for a variety of diagnostic and therapeutic purposes [63,64]. This method often leverages mitochondrial targeting moieties, such as triphenylphosphine [65]. In this investigation, nucleic acid-based nanocarriers equipped with Cyt C aptamers were developed to facilitate the targeted delivery of Cas12a/crRNA complexes to mitochondria. The experimental results demonstrated that InCasor successfully delivered these complexes into mitochondria by modulating the permeability of the mitochondrial membrane.

InCasor represents a breakthrough technology for the direct visualization of cells harboring mtDNA mutations in vivo, addressing a longstanding challenge. The ability to monitor mtDNA mutations provides critical insights into mtDNA heterogeneity and facilitates the detection of tumor tissues carrying specific mtDNA alterations. Future advancements will aim to expand InCasor’s applications to include nuclear genome analysis and the precise tracking of single nucleotide variant (SNV) sites with high spatiotemporal resolution. These efforts will further establish InCasor as a versatile tool for fundamental research on gene mutations, as well as for diagnostic and therapeutic applications involving in vivo gene editing.

By incorporating patient-specific antisense DNA sequences into an FNA nanostructure to selectively bind mutant mtRNA transcripts, this strategy could minimize off-target effects and ensures that the treatment is only activated in dysfunctional mitochondria. By designing an FNA nanostructure tailored to the patient’s unique mtDNA mutation profile, this approach provides high specificity, minimal off-target effects, and an adaptive therapeutic response. Over time, a shift in the heteroplasmy ratio towards wild-type mtDNA is expected, improving mitochondrial function and alleviating disease symptoms.

### 2.2. FNA Targeting Specific Enzymes in Mitochondria

Proteomic analyses reveal that mitochondria harbor approximately 1000 to 1500 distinct proteins, with only 13 encoded by mtDNA. The remaining proteins are encoded in nuclear DNA, synthesized in the cytoplasm, and subsequently imported into mitochondria [66]. This reliance underscores the importance of dynamic protein translocation across subcellular compartments for mitochondrial functionality. For example, various nucleases, crucial for mtDNA repair and mitochondrial RNA metabolism, are distributed between mitochondria and other organelles [67]. Human apurinic/apyrimidinic endonuclease 1 (APE1), a versatile enzyme involved in DNA repair, redox signaling, and transcription factor regulation [68,69], predominantly resides in the nucleus but can relocate to other compartments, such as mitochondria, under pathological conditions [70]. Developing a precise toolkit to monitor the intracellular dynamics of APE1 is therefore critical for advancing our understanding of disease mechanisms [71,72]. However, designing such a toolkit with the required subcellular accuracy remains a significant challenge.

**Figure 3:**
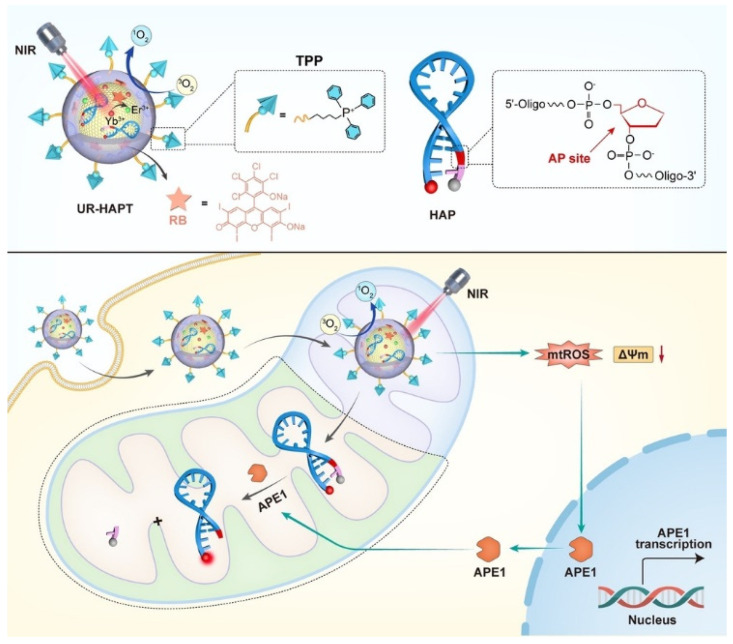
Schematic of the FNA nanosensor for in situ localization and NIR-light-activatable imaging of APE1 in mitochondria [73].

Inspired by this, Li’s group introduced a highly modular nanoplatform, UR-HAPT, capable of imaging subcellular APE1 dynamics in response to mitochondria-localized photodynamic therapy (PDT) activated by near-infrared (NIR) light (Figure 3) [73]. UR-HAPT is composed of four key elements: (1) an upconversion nanoparticle (UCNP) serving as a light transducer; (2) rose bengal (RB) functioning as a photosensitizer (PS); (3) triphenylphosphonium (TPP) acting as a mitochondria-targeting ligand; and (4) a DNA-based fluorescence reporter (HAP) designed for detecting APE1 enzymatic activity. The HAP probe incorporates an apurinic/apyrimidinic (AP) site within the stem of a molecular beacon, which is dual-labeled with the fluorophore Cy5 at the 3′-end and the quencher BHQ-2 at the 5′-end. This configuration ensures an exceptionally low fluorescence background through Förster resonance energy transfer (FRET) between Cy5 and BHQ-2. In the presence of APE1, cleavage at the AP site releases the quencher, resulting in a substantial fluorescence signal recovery. The TPP ligands on UR-HAPT facilitate precise mitochondrial targeting, while NIR light irradiation triggers the production of subcellular reactive oxygen species (ROS) via energy transfer from UCNPs to the PSs. Crucially, the HAP probe allows real-time tracking of APE1 translocation dynamics within mitochondria during the PDT process. This innovative design bridges the gap between photodynamic therapy and DNA-based biosensing technologies, enabling real-time subcellular molecular imaging during therapeutic interventions.

### 2.3. FNA Targeting Small Molecules and Metal Ions in Mitochondria

Mitochondria, often referred to as cellular powerhouses, generate ATP via oxidative phosphorylation [74]. However, during ATP production in cancer cells, abnormal accumulation of mitochondrial reactive oxygen species (ROS) has been observed [75,76]. Simultaneously, cancer cells synthesize large amounts of glutathione (GSH) within mitochondria to counteract oxidative stress caused by excessive ROS, thereby facilitating tumor progression [77,78]. Consequently, the disruption of energy metabolism and redox imbalance in mitochondria are widely recognized as defining characteristics of tumor development [79]. Investigating ATP and GSH—two critical molecules linked to mitochondrial energy metabolism and redox balance—through correlated imaging could shed light on their roles in tumorigenesis. While various DNA-based methods have been designed for the individual detection of ATP and GSH within cells [80,81], the simultaneous imaging of these two pivotal molecules remains an uncharted area of research.

**Figure 4:**
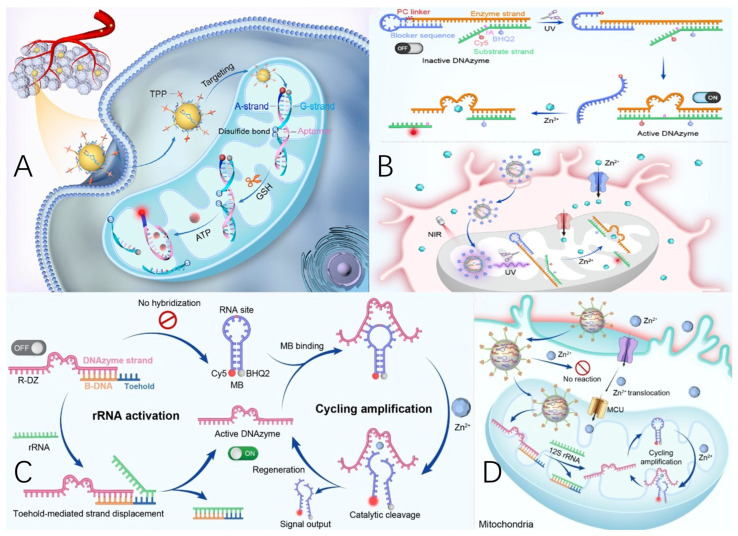
(**A**) Schematic of the design of a redox-activatable DNA nanodevice for AND-gated imaging of ATP and GSH in mitochondria [82]. (**B**) Schematic of the design and metal-ion sensing mechanism of L-DZ and the application of L-DZ/mUC for spatially selective imaging of [Zn^2+^]_m_ in Zn^2+^-induced neuropathology [83]. (**C**) The working principle of R-DZ/MB [84]. (**D**) The rRNA-regulated DNAzyme nanosensors for subcellular compartment-specific amplified imaging of Zn^2+^ in mitochondria [84].

Recently, Li’s group developed a novel approach that integrates a redox-sensitive aptamer sensor with nanoparticles engineered for precise targeting, enabling spatially resolved, AND-gated visualization of ATP and GSH within mitochondria [82]. As shown in Figure 4A, the A-G/NT system consists of two interconnected components: a redox-responsive aptamer probe (A-G) for dual molecular sensing and an organelle-specific nanoparticle (NP) for targeted mitochondrial delivery. The A-G probe was assembled by hybridizing two carefully designed DNA strands: the ATP-binding A-strand, derived from an aptamer, and the G-strand, which incorporates a strategically placed disulfide bond within its DNA backbone. This hybridization positioned Cy3 (attached to the A-strand) in close proximity to a black hole quencher (anchored to the G-strand), producing a low fluorescence background due to Förster resonance energy transfer (FRET). When GSH cleaves the disulfide bond in the G-strand, its binding affinity to the A-strand diminishes, reinstating the aptamer’s structure-switching ability to interact with ATP. Upon ATP recognition, the formation of an aptamer–ATP complex causes the cleaved G-strand to detach from the A-strand, leading to a fluorescence signal increase. Consequently, this stepwise activation process necessitates the concurrent presence of GSH and ATP to enable signal output, implementing an AND-gated mechanism for molecular imaging. Additionally, the A-G/NT design incorporates triphenylphosphonium (TPP) conjugation on the NPs, facilitating targeted delivery of the A-G probe for precise imaging within mitochondria [82].

Metal ions are indispensable for nearly all biological activities, with their precise distribution within cellular compartments being tightly controlled [85]. Investigating the localization and behavior of these ions at the subcellular level is crucial for understanding their roles in both normal physiological and disease-related mechanisms. For instance, mitochondria have been recognized as key reservoirs of Zn^2+^, and maintaining the equilibrium between mitochondrial Zn^2+^ ([Zn^2+^]_m_) and cytosolic Zn^2+^ ([Zn^2+^]_c_) is essential for regulating various processes, such as metabolic pathways and cellular signaling [86,87]. While numerous DNAzyme-based technologies have been devised to visualize metal ions in living cells [88,89,90], methodologies enabling in situ detection specifically within mitochondria remain underdeveloped, hindering further advancements in this area.

Li’s group presents a DNAzyme-based nanodevice tailored for the accurate imaging of subcellular metal ions within mitochondria [83]. As depicted in Figure 4B, the nanodevice, consists of two primary components: a UV light-responsive DNAzyme sensor probe (L-DZ) and a functionalized upconversion nanoparticle (UCNP) serving as a near-infrared (NIR) light-driven, organelle-specific delivery mechanism. The L-DZ probe was developed by strategically reconstructing the Zn^2+^-selective 17E DNAzyme. A blocker sequence was incorporated to form a hairpin structure that prevents the enzyme strand from hybridizing with the left arm of the substrate strand, thereby maintaining the DNAzyme in an inactive conformation. The substrate strand was modified with a Cy5 fluorophore and a black hole quencher (BHQ2) flanking the cleavage site, an adenosine ribonucleotide (rA). This configuration suppresses background fluorescence through Förster resonance energy transfer (FRET). Upon exposure to UV light, the photolysis of the photocleavable (PC) linker disrupts the hairpin structure, enabling the formation of active DNAzyme sensors. This activation transitions the DNAzyme from an OFF state to an ON state. In the presence of Zn^2+^, the substrate strand is cleaved, releasing a Cy5-labeled fragment, which produces a fluorescence signal indicative of metal-ion detection. To facilitate NIR-controlled activation, UCNPs were integrated into the sensor design. Additionally, the nanodevice was functionalized with triphenylphosphonium (TPP) for targeted mitochondrial delivery of L-DZ. Owing to its lipophilicity and strong positive charge, TPP is widely utilized for directing various cargos to mitochondria, leveraging the organelle’s negative membrane potential. The resulting L-DZ/mUC system enables precise visualization of Zn^2+^ within mitochondria by combining controlled localization with remote photoactivation, offering a robust platform for subcellular metal-ion imaging.

Li’s group proposed a ribosomal RNA-regulated DNAzyme sensor technology for spatially restricted imaging of Zn^2+^ within mitochondria [84]. As depicted in Figure 4C, the ribosomal RNA-regulated DNAzyme sensor (R-DZ/MB) comprises two components: an rRNA-activatable DNAzyme sensor module (R-DZ) and a molecular beacon-based signal amplification module. The R-DZ is engineered from the Zn^2+^-specific 17E DNAzyme, incorporating a blocker DNA sequence (B-DNA) with a toehold region [84]. The hybridization between the DNAzyme and B-DNA inhibits the enzyme strand from binding the substrate strand embedded in the MB loop, thus inactivating the DNAzyme’s catalytic function. The molecular beacon is designed with a cleavage site (adenosine ribonucleotide, rA) in the loop region and a fluorophore/quencher (Cy5/BHQ2) pair at the terminus of the extended stem, ensuring a low fluorescence background due to Förster resonance energy transfer. Upon addition of rRNA, the B-DNA forms a more stable duplex with rRNA via toehold-mediated strand displacement, releasing the active DNAzyme. In the presence of Zn^2+^, the DNAzyme cleaves the substrate molecular beacon, breaking it into two segments, leading to MB stem dehybridization and fluorescence recovery for metal ion sensing. The DNAzyme is then liberated from the molecular beacon segments due to reduced hybridization affinity and can participate in subsequent cycles of molecular beacon cleavage, achieving signal amplification via enzymatic multiple turnovers. Then, a mitochondria-targeted nanoparticle (mNP) was developed and combined with the 12S rRNA-responsive R-DZ/MB sensor to create a nanosensor, referred to as R-DZ/MB-mNP (Figure 4D). The sensing capability of R-DZ/MB is specifically activated by the mitochondria-exclusive 12S rRNA following the precise delivery of R-DZ/MB-mNP to the mitochondria, while showing no response to metal ions in regions outside the target. This design enables the nanosensor to achieve spatially specific and amplified imaging of metal ions within subcellular compartments by integrating organelle-targeting localization with rRNA-activated sensing mechanisms. Furthermore, the platform was employed to monitor mitochondrial Zn^2+^ fluctuations in real-time during ischemia and subsequent pharmacological treatment [84].

About overcoming mitochondrial membrane barriers, we concluded that the FNA nanostructures realize this mainly through the combination of membrane potential-driven accumulation, structural engineering, and biofunctional. First, mitochondria maintain a highly negative membrane potential (~−180 mV), which facilitates the accumulation of cationic moieties. FNA nanostructures are often conjugated with lipophilic cations, such as TPP, enabling efficient transport across the outer mitochondrial membrane and subsequent translocation into the inner mitochondrial membrane. Second, compact architectures of FNA nanostructures shield the negatively charged phosphate backbone, reducing electrostatic repulsion. Stimuli-responsive conformational changes, where FNA nanostructures undergo shape transformation or disassembly upon encountering mitochondrial microenvironmental cues, facilitate membrane penetration. Finally, beyond passive accumulation, FNA nanostructures can incorporate mitochondria-specific targeting ligands or peptide-based transporters that facilitate receptor-mediated endocytosis or direct mitochondrial translocation.

## 3. Therapeutics Based on FNA Target Mitochondria

Mitochondria play a crucial role in various cellular processes, including regulating cell proliferation and apoptosis, transmitting signals within cells, and maintaining the balance of cellular redox states. Disruptions in mitochondrial function can trigger a wide range of disorders, such as cancer, neurodegenerative diseases, cardiovascular conditions, and chronic inflammation [1,2,3]. Notably, the development of tumors demands significantly elevated ATP levels, which are heavily reliant on mitochondrial function. Consequently, mitochondria have become a promising focal point for the development of targeted cancer therapies [91]. A growing body of evidence suggests that therapeutic strategies aimed at mitochondria can optimize the therapeutic benefit by reducing the necessary drug dosage, overcoming resistance to multiple drugs, preventing tumor recurrence, and limiting metastasis, all while minimizing damage to surrounding healthy tissues [92,93,94]. In recent years, mitochondria-directed FNA nanostructures have garnered significant attention for their potential to enhance treatment effectiveness and precision. This form of therapy involves the targeted delivery of therapeutic compounds to the mitochondria [95] or directly modulating mitochondrial function to exert therapeutic effects [96,97,98].

### 3.1. FNA Nanostructure-Mediated Mitochondria-Targeted Therapeutics

Nucleic acid-based therapies, including antisense oligonucleotides (ASOs) and small interfering RNAs (siRNAs), have garnered significant interest for the treatment of a wide range of diseases. However, despite the growing approval of these therapeutic agents, effectively controlling and regulating their activity continues to present a significant challenge.

**Figure 5:**
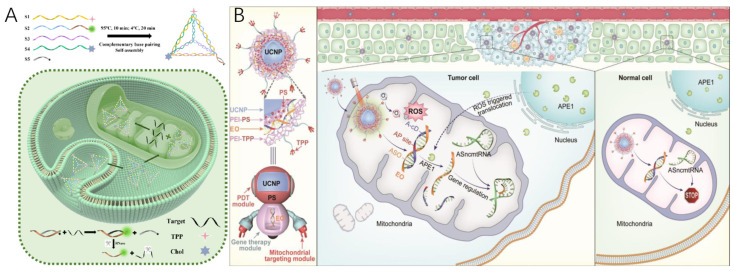
(**A**) Schematic of the composition process of TDFNs and TDFNs for miRNA-34a biomarker detection and AD therapy [99]. (**B**) Schematic of the design and structure of ASO/UCT and its application for spatially selective gene regulation and combinational tumor therapy [100].

In 2023, an FNA nanostructure, capable of self-assembling via complementary base pairing of designed single-stranded DNA, was developed by the Dong group [99]. This structure, a tetrahedron, was modified at three of its vertices with triphenylphosphine (TPP), cholesterol, and a functional ASO, forming what is referred to as tetrahedral DNA framework-based nanoparticles (TDFNs) (Figure 5A). These TDFNs have demonstrated the ability to cross the blood–brain barrier (BBB), penetrate neuronal cells, and target mitochondria. Additionally, the TDFNs are capable of recognizing miRNA-34a, generating fluorescence signals for Alzheimer’s disease diagnosis, while the incorporated ASO reduces miRNA-34a expression, modulating mitochondrial-associated apoptosis pathways and promoting the survival of neurons (Figure 5A).

The Li group presented a UCNP-based nanostructure for NIR light-controlled, enzymatically triggered regulation of gene expression and combinational tumor therapy [100]. As illustrated in Figure 5B, by integrating ASO with a UCNP-based mitochondria-targeted photodynamic therapy (PDT) platform. This nanostructure was designed to achieve precise, spatiotemporally-controlled gene regulation and combine tumor therapies, as follows: (1) the TPP-functionalized nanostructure ensures targeted localization to the mitochondria; (2) upon exposure to NIR light, the UCNPs emit green light, which excites photosensitizers to generate reactive oxygen species (ROS), leading to mitochondrial damage; (3) the ROS generated during PDT induces the accumulation of mitochondrial APE1, which activates EO, triggering the release of ASO to downregulate ASncmtRNAs in tumor cells, thereby facilitating on-demand gene therapy.

### 3.2. Mitochondria In Situ Self-Assembly of FNA Nanostructure for Therapeutics

The development of dynamically regulated supramolecular assembly systems within living cells to control cell fate represents a novel therapeutic approach [101]. To this end, various materials have been investigated for their ability to enable intracellular self-assembly, thereby modulating cellular functions [102,103,104]. For instance, nanostructures formed from short peptides have been shown to interfere with mitochondria and hold potential for therapeutic applications. In comparison to peptides, DNA molecules offer remarkable sequence programmability and predictable thermodynamic properties, making them superior candidates for the controlled assembly of dynamic nanostructures [9,10,11,12].

**Figure 6:**
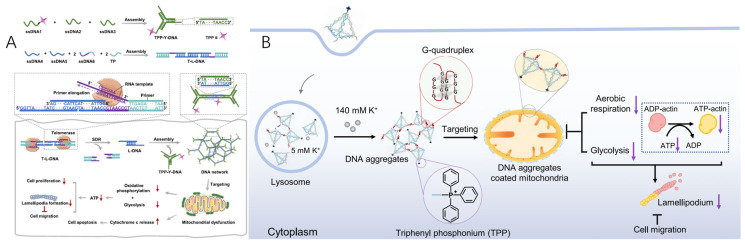
(**A**) Schematic of the assembly of TPP-Y-DNA and T-L-DNA and telomerase-mediated self-assembly of the DNA network inside cancer cells enabling mitochondrial interference and regulation of cellular behaviors [105]. The self-assembly of a DNA network within cancer cells, facilitated by telomerase activity, enables mitochondrial disruption and influences cellular functions. Telomerase, a distinctive ribonucleoprotein enzyme, is responsible for elongating telomeric DNA and extending telomeric primers. When telomerase is present, it catalyzes the primer extension in T-L-DNA, initiating strand displacement reactions and subsequently releasing L-DNA. The liberated L-DNA then hybridizes with TPP-Y-DNA through complementary base pairing, leading to the formation of an organized DNA network. (**B**) Schematic of the dynamic assembly of TDNs-G-TPP in living cells for mitochondrial interference and the consequent regulation of cellular behaviors [106].

In 2023, the Yao group designed an in situ-formed DNA-based network that self-assembled in cancer cells, specifically mediated by telomerase, realizing mitochondrial interference [105]. Two functional DNA modules were designed as depicted in Figure 6A: (i) The Y-shaped DNA (TPP-Y-DNA) was functionalized with TPP to target mitochondria. (ii) The telomerase-responsive linker DNA (T-L-DNA) was constructed with L-DNA and TP to recognize telomerase enzymes. Upon internalization by cancer cells, L-DNA was released from the T-L-DNA via a telomerase enzyme-induced SDR and subsequently hybridized with Y-DNA through base pairing, forming a DNA network. In contrast, this DNA network could not form in normal cells due to the absence of telomerase enzymes. The resulting DNA network encapsulated the mitochondria in cancer cells, disrupting the exchange of substances and inhibiting oxidative phosphorylation and glycolysis, leading to a reduction in ATP production [105]. The diminished ATP levels prevented the formation of the ATP–actin complex, thereby hindering lamellipodium formation, which is crucial for cell migration, and suppressing cancer cell proliferation. Additionally, a significant amount of cytochrome c (Cyt c) was released from the damaged mitochondria into the cytosol, triggering apoptosis in cancer cells.

The Yang group presented a K^+^-mediated dynamic assembly of DNA tetrahedrons for mitochondrial interference within living cells. One vertex of the DNA tetrahedron was functionalized with TPP for mitochondria targeting, while the other three vertices were conjugated with guanine-rich sequences to facilitate K^+^-induced aggregation of the tetrahedrons (Figure 6B) [106]. These DNA aggregates specifically localized to the mitochondria via TPP and disrupted mitochondrial functions, leading to a marked inhibition of cell migration. Investigation into the underlying mechanism revealed that the negatively charged DNA aggregates acted as a physical barrier on the mitochondria, obstructing substance exchange and consequently suppressing both aerobic respiration and glycolysis. This reduction in ATP production impaired the formation of the ATP–actin complex, thus inhibiting the development of lamellipodia, which are crucial for cellular movement.

## 4. Conclusions and Perspectives

The mentioned strategies based on FNA nanostructures targeting mitochondria are summarized in Table 1. As shown in Table 1, the strategies’ efficiency, biostability, off-target effects, and disadvantages are listed and compared. In conclusion, almost all FNA nanostructure-targeting strategies have high targeting efficiency and relatively good biostability. However, in some strategies, the off-target effect was not assessed by researchers even though it is actually a very critical indicator for evaluating the final targeting capability. By analyzing the disadvantages of these targeting strategies in Table 1, we conclude that the complex construction process is a common disadvantage of almost all targeting strategies based on FNA nanostructures. Meanwhile, some targeting strategies were triggered by light, but the light penetration depth in tissue is still in doubt.

This review summarizes and discusses recent progress in mitochondria-targeted molecular imaging and therapeutics facilitated by FNA nanostructures. By combining mitochondria-targeted nanodelivery approaches with various FNA-based sensing systems, spatially selective imaging of mitochondria-specific analytes has become achievable. For therapeutic applications, two primary strategies were highlighted. The first strategy involves the design of FNA nanodevices for the activatable delivery of therapeutic agents to mitochondria. For example, by combining pre-blocked ASO with a mitochondria-targeted photodynamic therapy (PDT) system, enzyme-activated gene regulation and combinatorial cancer therapy were achieved. Additionally, by tagging photosensitizers (PSs) and quenchers to the trans-cleavage substrate of the CRISPR-Cas12a system, PDT could be selectively triggered in the mitochondria of tumor cells with characteristic mtDNA mutations, further activating the cGAS–STING pathway for immunotherapy. The second strategy involves the use of conditionally controlled in situ FNA assembly to directly interfere with mitochondrial function in tumor cells.

Despite significant advancements in recent years, the field of mitochondria-targeted biosensing and therapy remains in its early stages, with numerous challenges yet to be addressed. The success of both mitochondrial sensing and therapeutic strategies heavily relies on the efficient delivery of engineered DNA components. Therefore, developing more advanced targeting approaches for the precise and effective delivery of functional DNA units to mitochondria continues to be a critical focus. For instance, lipid nanoparticles, commonly used in mRNA vaccines, could be engineered with mitochondria-targeting ligands to enhance the delivery of DNA systems [107]. TPP is currently the most widely used ligand for mitochondrial targeting, but other delocalized lipophilic cations, such as dequalinium, guanidine, and biguanide, offer additional possibilities for constructing mitochondria-targeted FNA nanostructures [108,109]. Furthermore, mitochondria-penetrating peptides (MPPs) and mitochondria-targeting sequences (MTSs) have not been fully explored in FNA nanostructure engineering, largely due to limitations in suitable modification methods. Peptide nucleic acids (PNAs) may serve as a promising solution, bridging this gap and facilitating the incorporation of MPPs and MTSs into mitochondria-targeted FNA nanostructures. Additionally, DNA origami nanostructures have emerged as a highly programmable and versatile platform for mitochondrial targeting, offering unprecedented precision in drug delivery, gene regulation, and biomolecular interactions. By leveraging the structural programmability and high cargo-loading capacity of DNA origami, researchers have developed mitochondria-targeting systems that can navigate the complex intracellular environment and overcome mitochondrial membrane barriers [110]. These nanostructures also can be functionalized with TPP to facilitate selective accumulation within mitochondria, thereby enhancing therapeutic efficacy while minimizing off-target effects. Meanwhile, some DNA origami platforms also can be used to study lipid transport between bilayers, which is relevant to mitochondrial and inter-organelle communication [111,112].

Mitochondria-targeted FNA nanostructures are designed to specifically localize in mitochondria and perform molecular imaging functions. However, these nanostructures may become irreversibly activated upon encountering their targets, leading to sensing before actual targeting occurs. To overcome this limitation, the light-activatable sensing approach was used for the programmable response of FNA nanostructures. Nevertheless, the light activation strategy still faces challenges due to the limited subcellular resolution of light. Given that several mitochondria-specific components have been identified, we hypothesize that these components could serve as endogenous mitochondrial triggers to regulate pre-blocked DNA probes, enabling precise subcellular imaging. For example, mtDNA sequences, which are uniquely localized in mitochondria, could act as stimuli to activate the sensing function of mitochondria-targeted biosensors. Additionally, most of the current mitochondria-targeted sensing methods have been validated primarily with well-known molecules. We are optimistic that these strategies can be effectively applied to uncover previously unexplored biological processes within mitochondria and in mitochondrial–organelle communications.

When applying FNA nanostructures for gene editing and mitochondria-targeted tumor or Alzheimer’s disease therapies, there is a potential risk of unintended effects on normal cells. Therefore, enhancing the target cell specificity of these mitochondria-targeted FNA systems is crucial. To address this challenge, intelligent FNA nanomachines, capable of responding and adapting to complex biological signals, hold promise as next-generation tools for mitochondria-targeted treatments. These smart FNA nanodevices can be meticulously engineered to incorporate cascade targeting mechanisms, thereby increasing targeting accuracy. For instance, DNA nanodevices can be equipped with tumor-specific ligands, such as aptamers that bind to overexpressed membrane proteins on tumor cells. Upon internalization into target cells, these devices could be directed to mitochondrial compartments using organelle-targeting ligands, allowing them to perform their therapeutic functions. Moreover, spatiotemporal control over the therapeutic actions of mitochondria-targeted systems provides an effective strategy for minimizing off-target effects. In this approach, the therapeutic activity of the DNA nanodevices is initially suppressed and later activated by tumor-specific triggers within mitochondria (such as reactive oxygen species or abnormal metabolite accumulation), thereby enhancing the selective targeting of cancer cells and reducing cytotoxicity to normal tissues. Additionally, with numerous ligands available for targeting different organelles, FNA nanostructures can be engineered to also target other cellular compartments, such as the nucleus or endoplasmic reticulum, for subcellular-specific imaging and therapies. Moving forward, more focus should be placed on designing intelligent FNA nanodevices that integrate both disease-specific recognition and organelle-targeting capabilities. Ultimately, we are optimistic that advancements in organelle-targeted FNA nanostructures will significantly contribute to both academic research and clinical applications.

## Figures and Tables

**Table 1:** Efficiency, biostability, off-target effect, and disadvantages of FNA nanostructure-based mitochondria-targeting strategies.

Strategies	Efficiency	Biostability	Off-Target Effect	Disadvantages
BPNS-hairpin DNA for mtRNA	High	Excellent	-	Dependence on dual-color imaging analysis instruments; without in vivo experiments
DNF-Cas12a/crRNA for mtRNA	High	Good	-	Off-target effect was not discussed
UR-HAPT for APE1	High	Excellent	Low	Limitation of light penetration depth in tissue; complex construction process
A-G/NT for ATP and GSH	High	Good	Low	Complex construction process
L-DZ/MUC for Zn^2+^	High	Good	Low	Complex construction process; without in vivo experiments
R-DZ/MB for Zn^2+^	High	Doubtful	-	Complex construction process; without in vivo experiments; signal attenuation in deep tissues in vivo
TDFNs for miRNA-34a	High	Excellent	Doubtful	Complex construction process
ASO/UCT for gene regulation	High	Excellent	Low	Limitation of light penetration depth in tissue; complex construction process
DNA network for ATP	High	Excellent	Low	Telomerase expression level differences affect the applicability of DNA network
TDNs-G-TPP	High	Excellent	Low	Affected by K^+^ concentration level differences

## Data Availability

Data will be made available on request.
